# Lean and Obese Zucker Rat Extensor Digitorum Longus Muscle high-frequency electrical stimulation (HFES) Data: Regulation of MAPKs Associated Proteins

**DOI:** 10.1016/j.dib.2017.11.054

**Published:** 2017-11-20

**Authors:** Kevin M. Rice, Anjaiah Katta, Nandini D.P.K. Manne, Ravikumar Arvapalli, Gautam K. Ginjupalli, Miaozong Wu, Shinichi Asano, Eric R. Blough

**Affiliations:** a Center for Diagnostic Nanosystems, Marshall University, Huntington, WV, USA; b Department of Internal Medicine, Joan C. Edwards School of Medicine, Marshall University, Huntington, WV, USA; c Biotechnology Graduate Program West Virginia State University, Institute, WV, USA; d Department of Health and Human Service, School of Kinesiology, Marshall University, Huntington, WV, USA; e Department of Public Heath, Marshall University, Huntington, WV, USA; f College of Health, Science, and Technology, University of Central Missouri, Warrensburg, MO, USA; g School of Education, Health, and Human Performance, Fairmont State University, Fairmont, WV, USA; h Department of Pharmaceutical Sciences and Research, School of Pharmacy, Marshall University, Huntington, WV, USA; i Department of Pharmacology, Physiology and Toxicology, Joan C. Edwards School of Medicine, Marshall University, Huntington, WV, USA

**Keywords:** Diabetes, Skeletal muscle, High-frequency electrical stimulation (HFES), Zucker rat, Extensor Digitorum Longus

## Abstract

Anaerobic exercise has been advocated as a prescribed treatment for the management of diabetes: however, alterations in exercise-induced signaling remain largely unexplored in the diabetic muscle. Here, we compare the basal and the in situ contraction-induced phosphorylation of the mitogen-activated protein kinases (MAPKs) ERK 1/2, p38, and JNK in the lean and obese (fa/fa) Zucker rat extensor digitorum longus (EDL) muscle following a single bout of contractile stimuli. This article represents data associated with prior publications from our (Katta et al., 2009a, 2009b, 2008) [1–3] and concurrent Data in Brief articles (Ginjupalli et al., 2017a, 2017b; Rice et al., 2017a, 2017b) [4–7].

**Specifications Table**

**Table:** 

Subject area	*Biology*
More specific subject area	*Diabetic skeletal muscle response to exercise*
Type of data	*Graph, figure*
How data was acquired	*Immunoblotting*
Data format	*Analyzed*
Experimental factors	*A high-frequency electrical stimulation (HFES) was used to produce 10 sets of 6 contractions over a 22-min period. Tissues were collected and protein was then isolated from tissue for western blot analysis.*
Experimental features	*EDL obtained from Lean and Obese male Zucker rats were used in this experiment*
Data source location	*Huntington, WV USA*
Data accessibility	*Data is with this article and is related to articles published and in review*[1], [2], [3], [4], [5], [6], [7].

**Value of the data**

• The data presented in this Brief is vital to understanding the effect of diabetes on skeletal muscle mechanotransduction.
• This data gives insight into the how diabetes alters tissue response to stimuli.
• This data provides a more thorough understanding of the MAPKs involvement in exercise mediated signaling in both diabetic and non-diabetic muscle tissue.

## 1 Data

### 1.1 ERK 1/2

**Fig. 1:**
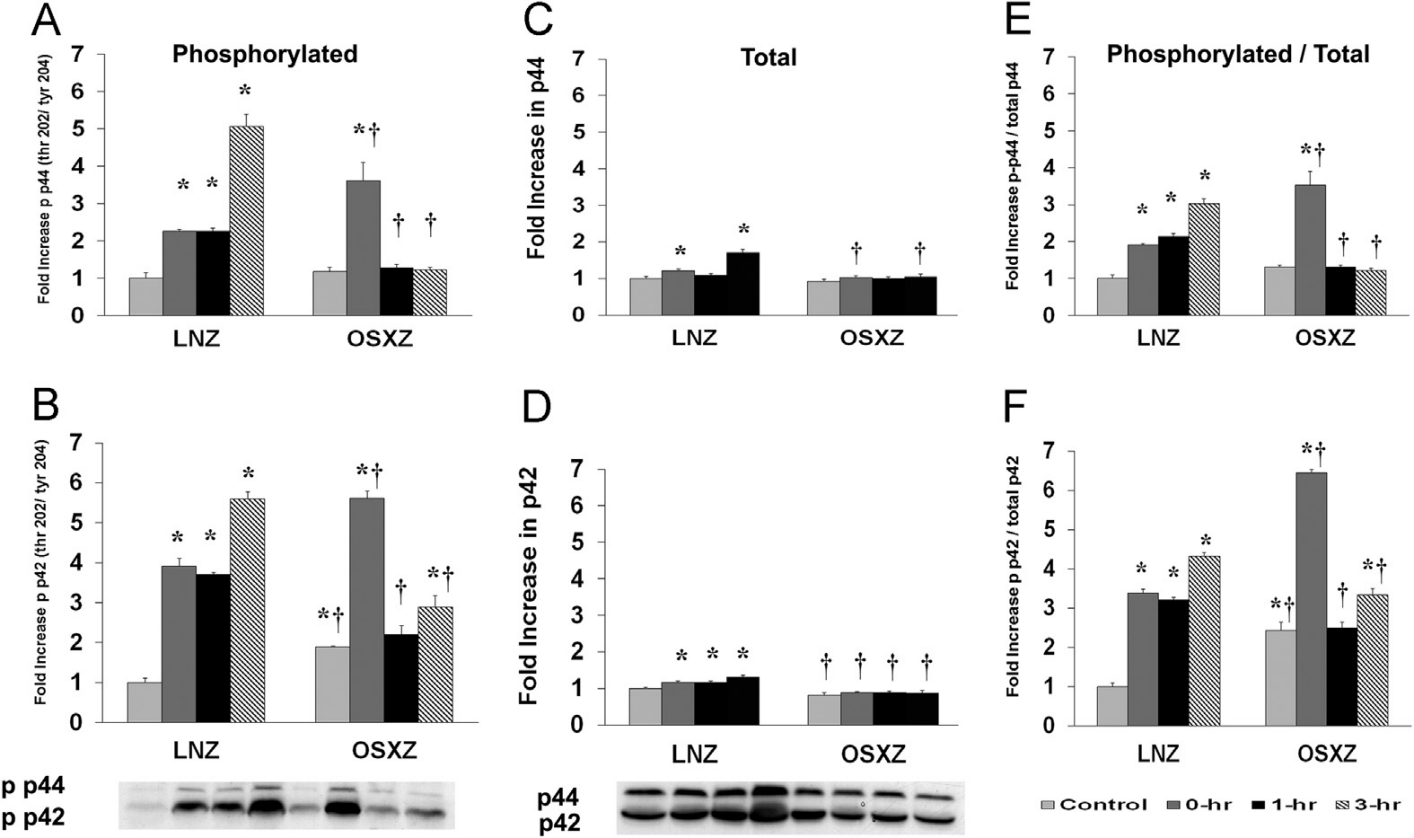
Diabetes alters HFES-induced expression and phosphorylation of p42/p44 MAPK rat EDL. The basal (control) and HFES-induced expression of p42/p44 in EDL from non-diabetic lean Zucker (LNZ) and diabetic obese syndrome X Zucker (OSXZ) rats. * Significantly different from HFES EDL within the same group (*p* < 0.05). † Significantly different from corresponding LNZ EDL (*p* < 0.05). *n* = 6/group.

To determine the effect of HFES on EDL from OSXZ and LNZ animals we evaluated the phosphorylation of ERK 1/2 at threonine 202 and tyrosine 204 (p44/p42 thr 202/tyr 204). EDL basal phosphorylation of p44 thr 202/tyr 204 demonstrated no significant difference in the OSXZ when compared to LNZ (Fig. 1A). HFES resulted in an increase in phosphorylation of p44 thr 202/tyr 204 in the LNZ EDL (126.9 ± 3.8%, 126.3 ± 4.1%, and 407.2 ± 31.6%, at 0, 1,and 3 h, *p* < 0.05) when compared to LNZ contralateral control (Fig. 1A). HFES resulted in an increase in phosphorylation of p44 thr 202/tyr 204 in the OSXZ EDL (242.7 ± 49.5% at 0 h, *p* < 0.05) when compared to OSXZ contralateral control (Fig. 1A). EDL basal phosphorylation of p42 thr 202/tyr 204 was higher (88.8 ± 2.7%, *p* < 0.05) in the OSXZ when compared to LNZ (Fig. 1B). HFES resulted in an increase in phosphorylation of p42 thr 202/tyr 204 in the LNZ EDL (290.6 ± 20.5%, 271.0 ± 4.1%, and 460.3 ± 16.7%, at 0, 1,and 3 h, *p* < 0.05) when compared to LNZ contralateral control (Fig. 1B). HFES resulted in an increase in phosphorylation of p42 thr 202/tyr 204 in the OSXZ EDL (371.9 ± 17.8% and 100.0 ± 28.4%, at 0 and 3 h, *p* < 0.05) when compared to OSXZ contralateral control (Fig. 1B).

To determine the effect of HFES on EDL from diabetic male obese syndrome-X Zucker (OSXZ) diabetic and nondiabetic male normal lean Zucker (LNZ) animals we evaluated the expression of extracellular-signal-regulated kinase (ERK 1/2 – p44/p42). EDL basal p44 content demonstrated no significant difference in the OSXZ when compared to LNZ (Fig. 1C). HFES resulted in an increase in p44 in the LNZ EDL (21.6 ± 4.2% and 70.8 ± 9.5%, at 0, and 3 h, *p* < 0.05) when compared to LNZ contralateral control (Fig. 1C). However, HFES did not elicit a response in the OSZX EDL when compared to contralateral OXSZ control (Fig. 1C). EDL basal p42 content was lower (19.0 ± 7.9%, *p* < 0.05) in the OSXZ when compared to LNZ (Fig. 1D). HFES resulted in an increase in p42 in the LNZ EDL (15.7 ± 3.2%, 16.7 ± 3.4%, and 30.8 ± 4.6%, at 0, 1,and 3 h, *p* < 0.05) when compared to LNZ contralateral control (Fig. 1D). However, HFES did not elicit a response in the OSZX EDL when compared to contralateral OXSZ control. EDL basal p44 content demonstrated no significant difference in the OSXZ when compared to LNZ (Fig. 1D). HFES resulted in an increase in p44 in the LNZ EDL (21.6 ± 4.2% and 70.8 ± 9.5%, at 0, and 3 h, *p* < 0.05) when compared to LNZ contralateral control. However, HFES did not elicit a response in the OSZX EDL when compared to contralateral OXSZ control (Fig. 1D).

To determine the effect of HFES on EDL from OSXZ and LNZ animals we evaluated the phosphorylation of ERK 1/2 (p44/p42 thr 202/tyr 204) to total p42/p44. EDL basal phosphorylation of p44 thr 202/tyr 204 to total p44 demonstrated no significant difference in the OSXZ when compared to LNZ (Fig. 1E). HFES resulted in an increase in phosphorylation of p44 thr 202/tyr 204 to total p44 in the LNZ EDL (90.5 ± 3.7%, 113.7 ± 8.0%, and 203.2 ± 13.1%, at 0, 1,and 3 h, *p* < 0.05) when compared to LNZ contralateral control (Fig. 1E). HFES resulted in an increase in phosphorylation of p44 thr 202/tyr 204 to total p44 in the OSXZ EDL (222.4 ± 36.7% at 0 h, *p* < 0.05) when compared to OSXZ contralateral control (Fig. 1E). EDL basal phosphorylation of p42 thr 202/tyr 204 to total p42 was higher (143.0 ± 20.5%, *p* < 0.05) in the OSXZ when compared to LNZ (Fig. 1F). HFES resulted in an increase in phosphorylation of p42 thr 202/tyr 204 to total p42 in the LNZ EDL (239.2 ± 8.7%, 221.1 ± 5.9%, and 332.3 ± 9.0%, at 0, 1,and 3 h, *p* < 0.05) when compared to LNZ contralateral control (Fig. 1F). HFES resulted in an increase in phosphorylation of p42 thr 202/tyr 204 to total p42 in the OSXZ EDL (402.5 ± 7.4% and 90.9 ± 15.6%, at 0 and 3 h, *p* < 0.05) when compared to OSXZ contralateral control (Fig. 1F).

### 1.2 P38

**Fig. 2:**
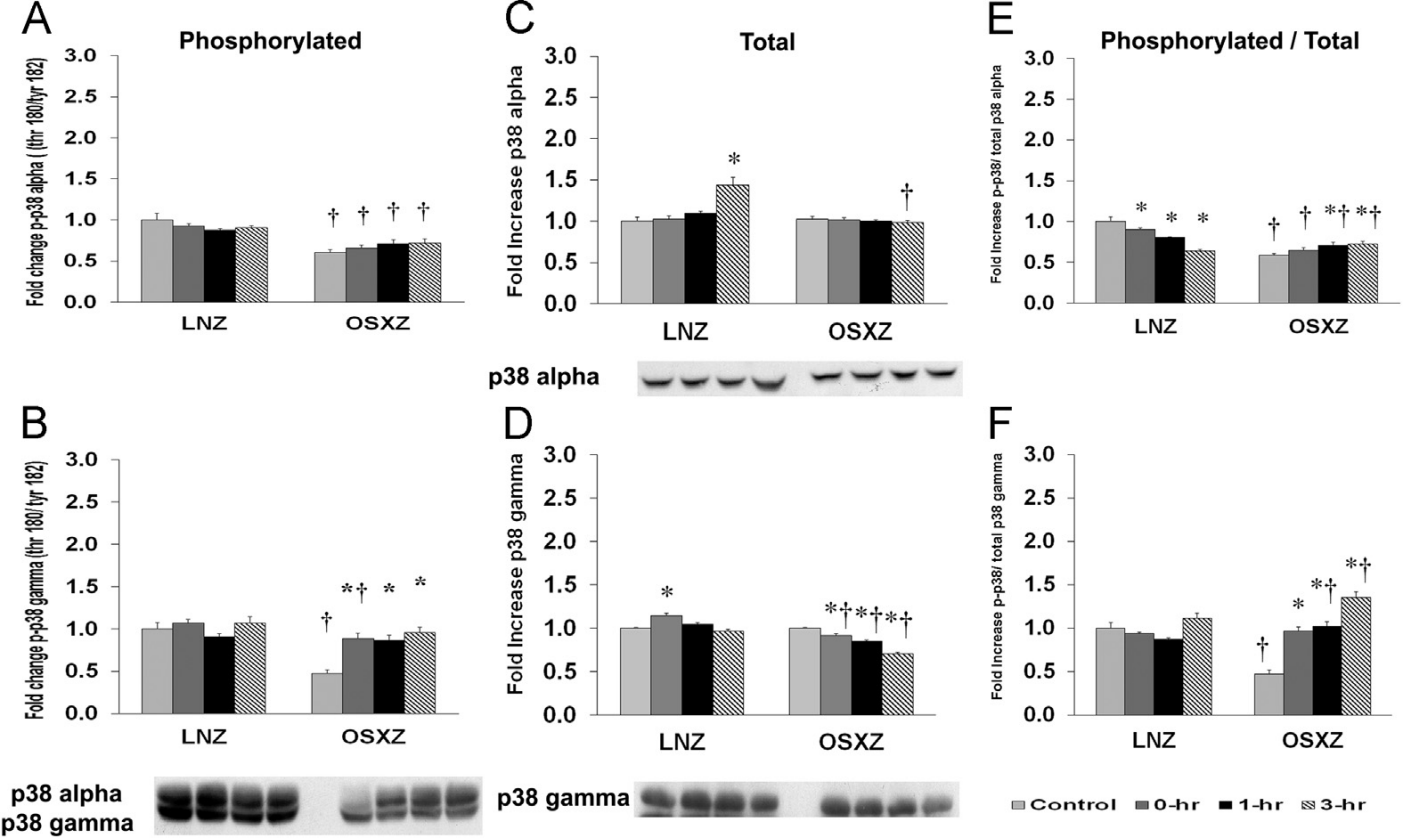
Diabetes alters l HFES-induced expression and phosphorylation of p38 MAPK rat EDL. The basal (control) and HFES-induced expression of p38 in EDL from non-diabetic lean Zucker (LNZ) and diabetic obese syndrome X Zucker (OSXZ) rats. * Significantly different from HFES EDL within the same group (*p* < 0.05). † Significantly different from corresponding LNZ EDL (*p* < 0.05). *n* = 6/group.

To determine the effect of HFES on EDL from OSXZ and LNZ animals we evaluated the phosphorylation of p39 alpha and gamma at threonine 180 and tyrosine 182 (p38 thr 180/tyr 182). EDL basal phosphorylation of p38 alpha thr 180/tyr 182 was lower (39.8 ± 3.9%, *p* < 0.05) in the OSXZ when compared to LNZ. HFES did not elicit a response in phosphorylation of p38 alpha thr 180/tyr 182 in the LNZ EDL when compared to LNZ contralateral control (Fig. 2A). HFES did not elicit a response in OSXZ EDL when compared to OSXZ contralateral control (Fig. 2A). EDL basal phosphorylation of p38 gamma thr 180/tyr 182 was lower (52.7 ± 4.5%, *p* < 0.05) in the OSXZ when compared to LNZ (Fig. 2B). HFES did not elicit a response in phosphorylation of p38 gamma thr 180/tyr 182 in the LNZ EDL when compared to LNZ contralateral control (Fig. 2B). HFES resulted in an increase in phosphorylation of p38 gamma thr 180/tyr 182 in the OSXZ EDL (41.6 ± 5.8%, 39.4 ± 5.4%, and 48.1 ± 6.2%, at 0, 1,and 3 h, *p* < 0.05) when compared to OSXZ contralateral control (Fig. 2B).

To determine the effect of HFES on EDL from OSXZ and LNZ animals we evaluated the expression of p38 alpha and gamma. EDL basal p38 alpha content demonstrated no significant difference in the OSXZ when compared to LNZ. HFES resulted in an increase in p38 alpha in the LNZ EDL (43.8 ± 8.8%, at 3 h, *p* < 0.05) when compared to LNZ contralateral control (Fig. 2C). However, HFES did not elicit a response in the OSZX EDL when compared to contralateral OXSZ control (Fig. 2C). EDL basal p38 gamma content demonstrated no significant difference in the OSXZ when compared to LNZ. HFES resulted in an increase in p38 gamma in the LNZ EDL (14.1 ± 2.9%, at 0 h, *p* < 0.05) when compared to LNZ contralateral control (Fig. 2D). However HFES resulted in an decrease in p38 gamma (8.0 ± 1.8%, 14.4 ± 0.9%, and 29.2 ± 1.4%, at 0, 1,and 3 h, *p* < 0.05) in the OSZX EDL when compared to contralateral OXSZ control (Fig. 2D).

To determine the effect of HFES on EDL from OSXZ and LNZ animals we evaluated the phosphorylation of p38 alpha and gamma thr 180/tyr 182 to total p38 alpha and gamma. EDL basal phosphorylation of p38 alpha thr 180/tyr 182 to total p38 alpha was higher (41.5 ± 2.4%, *p* < 0.05) in the OSXZ when compared to LNZ (Fig. 2E). HFES resulted in an decrease in phosphorylation of p38 alpha thr180/tyr 182 to total p38 alpha in the LNZ EDL (19.4 ± 0.6%, 36.2 ± 2.2%, and 41.5 ± 2.4%, at 0, 1,and 3 h, *p* < 0.05) when compared to LNZ contralateral control (Fig. 2E). HFES resulted in an increase in phosphorylation of p38 thr 180/tyr 182 to total p38 alpha in the OSXZ EDL (12.3 ± 3.9% and 13.9 ± 3.8%, at 1 and 3 h, *p* < 0.05) when compared to OSXZ contralateral control (Fig. 2E). EDL basal phosphorylation of p38 gamma thr 180/tyr 182 to total p38 gamma demonstrated a significant decrease (52.5 ± 4.2%, *p* < 0.05) in the OSXZ when compared to LNZ (Fig. 2F). HFES did not elicit a response in phosphorylation of p38 gamma thr 180/tyr 182 to total p38 gamma in the LNZ EDL when compared to LNZ contralateral control (Fig. 2F). HFES resulted in an increase in phosphorylation of p38 gamma thr 180/tyr 182 total p38 in the OSXZ EDL (49.3 ± 4.2%, 54.2 ± 4.7%, and 87.8 ± 6.7%, at 0, 1, and 3 h, *p* < 0.05) when compared to OSXZ contralateral control (Fig. 2F).

### 1.3 JNK

**Fig. 3:**
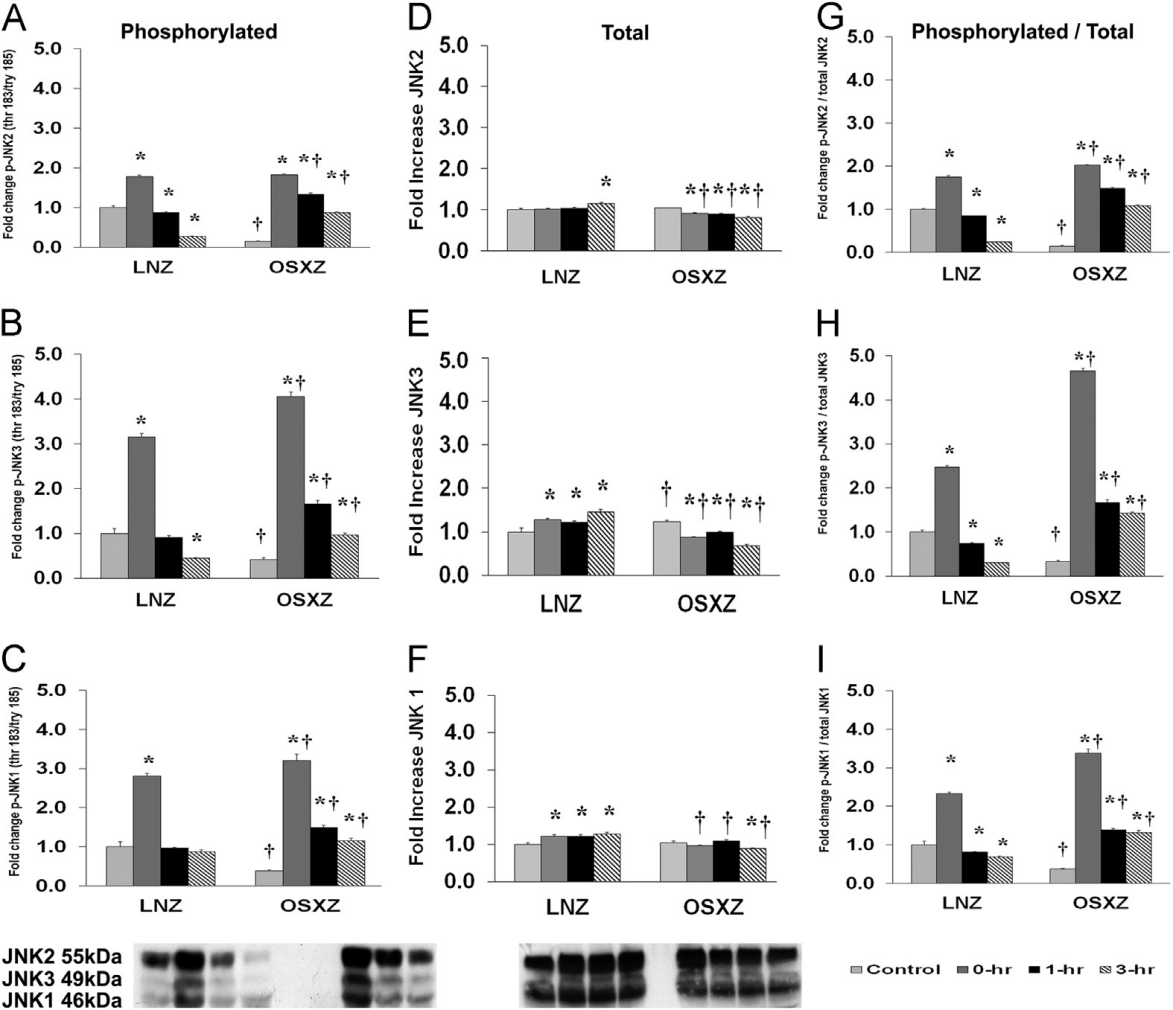
Diabetes alters l HFES-induced expression and phosphorylation of JNK MAPK rat EDL. The basal (control) and HFES-induced expression of JNK in EDL from non-diabetic lean Zucker (LNZ) and diabetic obese syndrome X Zucker (OSXZ) rats. * Significantly different from HFES EDL within the same group (*p* < 0.05). † Significantly different from corresponding LNZ EDL (*p* < 0.05). *n* = 6/group.

To determine the effect of HFES on EDL from OSXZ and LNZ animals we evaluated the phosphorylation of JNK 1/2/3 at threonine 183 and tyrosine 185 (JNK 1/2/3 thr 180/tyr 182). EDL basal phosphorylation of JNK2 thr 183/tyr 185 was lower (84.9 ± 1.9%, *p* < 0.05) in the OSXZ when compared to LNZ (Fig. 3A). HFES resulted in an increase (79.1 ± 3.4%, at 0 h, *p* < 0.05) and a decrease (12.1 ± 2.3%, and 72.3 ± 2.0%, at 1, and 3 h, *p* < 0.05) in phosphorylation of JNK2 thr 183/tyr 185 in the LNZ EDL when compared to LNZ contralateral control (Fig. 3A). HFES resulted in an increase (168.3 ± 1.9%, 118.5 ± 4.0%, and 73.2 ± 1.6%, at 0, 1,and 3 h, *p* < 0.05) in phosphorylation of JNK2 thr 183/tyr 185 in the OSXZ EDL when compared to OSXZ contralateral control (Fig. 3A). EDL basal phosphorylation of JNK3 thr 183/tyr 185 was lower (58.2 ± 3.6%, *p* < 0.05) in the OSXZ when compared to LNZ (Fig. 3B). HFES resulted in an increase (215.8 ± 7.3%, at 0 h, *p* < 0.05) and a decrease (55.5 ± 1.3%, at 3 h, *p* < 0.05) in phosphorylation of JNK3 thr 183/tyr 185 in the LNZ EDL when compared to LNZ contralateral control (Fig. 3B). HFES resulted in an increase (363.9 ± 9.8%, 123.7 ± 8.5%, and 54.8 ± 3.9%, at 0, 1,and 3 h, *p* < 0.05) in phosphorylation of JNK3 thr 183/tyr 185 in the OSXZ EDL when compared to OSXZ contralateral control (Fig. 3B). EDL basal phosphorylation of JNK1 thr 183/tyr 185 was lower (62.0 ± 3.0%, *p* < 0.05) in the OSXZ when compared to LNZ (Fig. 3C). HFES resulted in an increase (180.6 ± 7.7%, at 0 h, *p* < 0.05) in phosphorylation of JNK1 thr 183/tyr 185 in the LNZ EDL when compared to LNZ contralateral control (Fig. 3C). HFES resulted in an increase (283.168 ± 15.2%, 111.1 ± 5.8%, and 77.4 ± 6.8%, at 0, 1, and 3 h, *p* < 0.05) in phosphorylation of JNK1 thr 1830/tyr 185 in the OSXZ EDL when compared to OSXZ contralateral control (Fig. 3C).

To determine the effect of HFES on EDL from OSXZ and LNZ animals we evaluated the expression of c-Jun N-terminal kinase (JNK 1/2/3) MAPK. EDL basal JNK2 content demonstrated no significant difference in the OSXZ when compared to LNZ (Fig. 3D). HFES resulted in an increase in JNK2 in the LNZ EDL (145.0 ± 3.3% at 3 h, *p* < 0.05) when compared to LNZ contralateral control (Fig. 3D). However HFES resulted in a decrease in JNK2 in the OSXZ EDL (13.3 ± 1.4%, 14.0 ± 1.1%, and 22.7 ± 2.6%, at 0, 1,and 3 h, *p* < 0.05) in the OSZX EDL when compared to contralateral OXSZ control (Fig. 3D). EDL basal JNK3 content demonstrated a significant increase (22.8 ± 3.7%, *p* < 0.05) in the OSXZ when compared to LNZ (Fig. 3E). HFES resulted in an increase in JNK3 in the LNZ EDL (27.6 ± 2.7%, 21.8 ± 3.6%, and 46.1 ± 4.9%, at 0, 1, and 3 h, *p* < 0.05) when compared to LNZ contralateral control (Fig. 3E). However HFES resulted in an decrease in JNK3 (35.8 ± 1.8%, 23.8 ± 1.6%, and 55.2 ± 3.0%, at 0, 1, and 3 h, *p* < 0.05) in the OSZX EDL when compared to contralateral OXSZ control (Fig. 3E). EDL basal JNK1 content demonstrated no significant difference in the OSXZ when compared to LNZ (Fig. 3F). HFES resulted in an increase in JNK1 in the LNZ EDL (22.1 ± 4.4%, 21.1 ± 5.4%, and 28.5 ± 4.4%, at 0, 1,and 3 h, *p* < 0.05) when compared to LNZ contralateral control (Fig. 3F). However HFES resulted in a decrease in JNK1 in the OSXZ EDL (15.9 ± 1.8%, at 3 h, *p* < 0.05) in the OSZX EDL when compared to contralateral OXSZ control (Fig. 3F).

To determine the effect of HFES on EDL from diabetic male OSXZ and LNZ animals we evaluated the phosphorylation of JNK 1/2/3 thr 183/tyr 185 to total JNK 1/2/3. EDL basal phosphorylation of JNK2 thr 183/tyr 185 to total JNK2 was lower (85.7 ± 1.7%, *p* < 0.05) in the OSXZ when compared to LNZ (Fig. 3G). HFES resulted in an increase (75.6 ± 2.6%, at 0 h, *p* < 0.05) and decrease (14.9 ± 0.9%, and 75.9 ± 1.1%, at 1, and 3 h, *p* < 0.05) in phosphorylation of JNK2 thr 183/tyr 185 to total JNK2 in the LNZ EDL when compared to LNZ contralateral control (Fig. 3G). HFES resulted in an increase (187.8 ± 1.4%, 133.9 ± 2.7%, and 94.4 ± 21%, at 0, 1, and 3 h, *p* < 0.05) in phosphorylation of JNK2 thr 183/tyr 185 to total JNK2 alpha in the OSXZ EDL when compared to OSXZ contralateral control (Fig. 3G). EDL basal phosphorylation of JNK3 thr 183/tyr 185 to total JNK3 was lower (66.2 ± 1.9%, *p* < 0.05) in the OSXZ when compared to LNZ (Fig. 3H). HFES resulted in an increase (147.3 ± 3.3%, at 0 h, *p* < 0.05) and decrease (25.8 ± 1.9%, and 69.5 ± 0.8%, at 1, and 3 h, *p* < 0.05) in phosphorylation of JNK3 thr 183/tyr 185 to total JNK3 in the LNZ EDL when compared to LNZ contralateral control (Fig. 3H). HFES resulted in an increase (431.8 ± 6.0%, 133.0 ± 6.0%, and 109.3 ± 2.07%, at 0, 1, and 3 h, *p* < 0.05) in phosphorylation of JNK3 thr 183/tyr 185 to total JNK3 alpha in the OSXZ EDL when compared to OSXZ contralateral control (Fig. 3H). EDL basal phosphorylation of JNK1 thr 183/tyr 185 to total JNK1 was lower (63.2 ± 1.6%, *p* < 0.05) in the OSXZ when compared to LNZ (Fig. 3I). HFES resulted in an increase (133.0 ± 3.6%, at 0 h, *p* < 0.05) and decrease (18.9 ± 1.9%, and 31.3 ± 1.9%, at 1, and 3 h, *p* < 0.05) in phosphorylation of JNK1 thr 183/tyr 185 to total JNK1 in the LNZ EDL when compared to LNZ contralateral control (Fig. 3I). HFES resulted in an increase (301.0 ± 10.8%, 101.8 ± 3.5%, and 95.4 ± 5.8%, at 0, 1, and 3 h, *p* < 0.05) in phosphorylation of JNK1 thr 183/tyr 185 to total JNK1 in the OSXZ EDL when compared to OSXZ contralateral control (Fig. 3I).

## 2 Experimental design, materials and methods

### 2.1 Animals

All procedures were conducted in strict accordance with the Guide for the Care and Use of Laboratory Animals as approved by the Council of the American Physiological Society and the Animal Use Review Board of Marshall University. Young (10 week, *n* = 12) male lean Zucker (non-diabetic) (LNZ) and young (10 week, *n* = 12) male obese syndrome-X Zucker (diabetic) (OSXZ) rats were obtained from the Charles River Laboratories and barrier housed one per cage in an AAALAC approved vivarium. Housing conditions consisted of a 12H: 12H dark-light cycle and the temperature was maintained at 22 ± 2 °C. Animals were provided food and water *ad libitum*. Rats were allowed to recover from shipment for at least two weeks before the commencement of experimentation during which time the animals were carefully observed and weighed weekly.

### 2.2 Materials

Anti-Erk1/2 (p42/p44) (#9102), phospho-Erk1/2 (P42/p44) Thr202/Tyr204 (#9106), p38 (#9212), phospho-p38 Thr180/Tyr182 (#9216), SAPK/Jnk (#9252), phospho-SAPK /JnkThr183/Tyr185 (#9251), Mouse IgG, and Rabbit IgG antibodies were purchased from Cell Signaling Technology (Beverly, MA). Enhanced chemiluminescence (ECL) western blotting detection reagent was from Amersham Biosciences (Piscataway, NJ). Precast 10% and 15% SDS-PAGE gels were purchased from Lonza (Rockland, ME). Enhanced chemiluminescence (ECL) western blotting detection reagent was purchased from Amersham Biosciences (Piscataway, NJ). Restore western blot stripping buffer was obtained from Thermo scientific (Rockford, IL) and 3T3 cell lysates from Santa Cruz Biotechnology (Santa Cruz, CA). All other chemicals were from Sigma (St. Louis, MO).

### 2.3 Contractile stimulation of skeletal muscles

The high-frequency electrical stimulation (HFES) model has been previously described [8] and was chosen on the basis of its efficacy in stimulating protein translation and muscle hypertrophy in vivo [9]. The HFES model used in the present study produced 10 sets of 6 contractions with an overall protocol time of 22 min. Animals were killed by a lethal dose of pentobarbital sodium at baseline, immediately following, 1 h or 3 h (*n* = 6 normal, *n* = 6 diabetic for 0, 1, and 3 h) after HFES. Once excised, muscles were blotted dry, trimmed of visible fat and tendon projections, weighed, immediately frozen in liquid nitrogen, and stored at − 80 °C.

### 2.4 Immunoblot analysis

Skeletal muscles were snap-frozen in liquid nitrogen at the end of each experiment. Protein isolates were prepared from the muscles by pulverizing the samples under liquid nitrogen using a mortar and pestle before washing (3 × 5 min) with ice-cold phosphate buffered saline (PBS). T-PER (2 mL/1 g tissue weight) (Pierce, Rockford, IL) supplemented with 100 mM NaF, 1 mM Na_3_VO_4_, 2 mM PMSF 1 μg/ml aprotinin, 1 μg/ml leupeptin, and 1 μg/ml pepsatin was used to extract proteins as detailed by the manufacturer. After centrifugation (1000*g* × 10 min), the supernatant was collected and the protein concentration of the homogenates was determined in triplicate using the Bradford method (Pierce) with bovine serum albumin as a standard. Samples were diluted to a concentration of 1.5 mg/mL in SDS-loading buffer and boiled for 5 min before loading thirty micrograms of total protein for separation on 10% or 15% SDS-PAGE gels. After electrophoresis, proteins were transferred onto Hybond nitrocellulose membranes (Amersham Biosciences, Piscataway, NJ) using standard conditions and stained with Ponceau S to verify transfer and equal loading of lanes.

Membranes were blocked in buffer (5% nonfat dry milk in TBST) for 1 h at room temperature, washed (TBST, 3 × 5 min), and incubated in primary antibody overnight at 4 °C. Unbound antibody was removed by washing the membranes (TBST, 3 × 5 min) and the membranes were incubated in horseradish peroxidase (HRP)-linked secondary antibodies for 1 h at room temperature and rewashed (TBST, 3 × 5 min).

The exposure time was adjusted to keep the integrated optical densities within a linear and non-saturated range. Molecular weight markers (Cell Signaling) were used as mass standards and NIH 3T3 cell lysates were included as positive controls. Membranes were stripped using the Restore western blot stripping buffer as detailed by the manufacturer. The absence of antibody binding after stripping was confirmed using the ECL reagent before washing (TBST, 3 × 5 min) the membranes and reprobing. Experimental error associated with membrane stripping and reprobing was minimized by randomizing the antibody incubations between experiments.

### 2.5 Data analysis

Data were analyzed using Sigma Stat 3.0 statistical software and the results are presented as mean ± SEM. Two-way ANOVA followed by the Student-Newman-Keuls post-hoc testing to determine differences between groups. The level of significance accepted *a priori* was < 0.05.
